# ASPM promotes homologous recombination-mediated DNA repair by safeguarding BRCA1 stability

**DOI:** 10.1016/j.isci.2021.102534

**Published:** 2021-05-12

**Authors:** Shibin Xu, Xingxuan Wu, Peipei Wang, Sheng-Li Cao, Bin Peng, Xingzhi Xu

**Affiliations:** 1 College of Life Sciences, Capital Normal University, Beijing 100048, China; 2 Department of Chemistry, Capital Normal University, Beijing 100048, China; 3 Guangdong Key Laboratory for Genome Stability & Disease Prevention and Marshall Laboratory of Biomedical Engineering, Shenzhen University School of Medicine, Shenzhen, Guangdong 518060, China; 4 Shenzhen University-Friedrich Schiller Universität Jena Joint PhD Program in Biomedical Sciences, Shenzhen University School of Medicine, Shenzhen, Guangdong 518060, China; 5 International Cancer Center, Shenzhen University School of Medicine, Shenzhen, Guangdong 518060, China

**Keywords:** Molecular biology, Cell biology, Functional aspects of cell biology

## Abstract

DNA double-strand break (DSB) repair by homologous recombination (HR) is essential for ensuring genome stability. *Abnormal spindle-like microcephaly-associated* (*ASPM*) gene encodes a spindle protein that is commonly implicated in primary microcephaly. We found that ASPM is recruited to sites of DNA damage in a PARP2-dependent manner. ASPM interacts with BRCA1 and its E3 ligase HERC2, preventing HERC2 from accessing to BRCA1 and ensuring BRCA1 stability. Inhibition of ASPM expression promotes HERC2-mediated BRCA1 degradation, compromises HR repair efficiency and chromosome stability, and sensitizes cancer cells to ionizing radiation. Moreover, we observed a synergistic effect between ASPM and PARP inhibition in killing cancer cells. This research has uncovered a novel function for ASPM in facilitating HR-mediated repair of DSBs by ensuring BRCA1 stability. ASPM might constitute a promising target for synthetic lethality-based cancer therapy.

## Highlights

• ASPM is recruited to sites of DNA damage in a PARP2-dependent manner.
• ASPM promotes DSB-end resection to facilitate HR repair.
• ASPM prevents HERC2 from accessing to BRCA1 and ensuring BRCA1 stability.
• Inhibition of ASPM sensitizes cancer cells to ionizing radiation and PARP inhibitor.

## Graphical abstract

**Figure:**
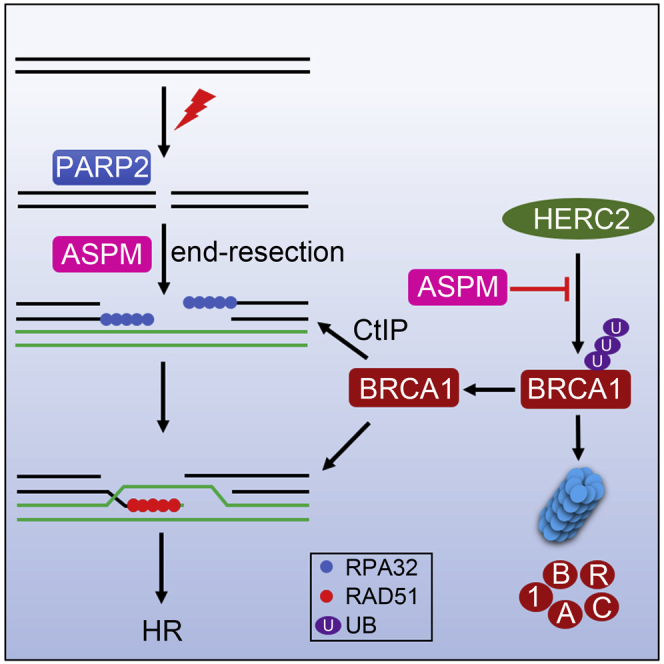

## Introduction

Microcephaly primary hereditary (MCPH) is a rare autosomal recessive genetic disease characterized by neurodevelopmental defects. MCPH can result from mutations in at least 25 genes, while mutations in the *abnormal spindle-like microcephaly-associated* (*ASPM*) gene (Bond et al., 2002) are the most common cause, representing ~40% of all MCPH cases (Jean et al., 2020). We are among the first to report that (1) both ASPM and microcephalin (MCPH1) are centrosomal/spindle proteins (Zhong et al., 2005, 2006); (2) both proteins functionally associate with breast cancer type 1 susceptibility protein BRCA1 (Xu et al., 2004; Zhong et al., 2005); (3) MCPH1 plays an important role in DNA damage-induced cellular responses (Xu et al., 2004).

BRCA1 is highly expressed in the embryonic neurepithelium and adult neurogenic areas (Korhonen et al., 2003). The development of central nervous system-specific *Brca1* knockout mice helped demonstrate that *Brca1* is required for embryonic neurodevelopment by preventing *Atm/p53*-dependent apoptosis of early neural progenitors (Pao et al., 2014; Pulvers and Huttner, 2009); however, centrosome-associated phenotypes observed in these animals (such as cell polarity) were independent of the Atm/p53 axis (Pao et al., 2014). Nevertheless, BRCA1 is essential for mediating DNA repair by homologous recombination (HR). BRCA1 directly interacts with Partner and localizer of BRCA2 (PALB2), fine-tuning HR repair partly by modulating the PALB2-dependent loading of the BRCA2-RAD51 repair machinery at DNA breaks (Sy et al., 2009). BRCA1 is also one of 22 causative genes of Fanconi anemia (FA), a rare autosomal or x-chromosomal recessive human genetic disease (Fang et al., 2020). Typical FA-like features include a short stature, microcephaly, skin hyperpigmentation. At the molecular level, the FA pathway mediates interstrand crosslink repair via HR (Fang et al., 2020).

BRCA1 protein stability is regulated by the E3 ligase HERC2 (Wu et al., 2010). In response to DNA damage, RNF8-dependent HERC2 recruitment to the damage site stabilizes RNF168 and promotes K63 polyubiquitination in H2A-type histones flanking DSBs, promoting HR repair (Bekker-Jensen et al., 2010; Danielsen et al., 2012). Naturally occurring and targeted *Herc2* mutant mice, as well as human homozygous or compound heterozygous *HERC2* variants exhibit global neurodevelopmental disability (Abraham et al., 2019; Ji et al., 1999; Lehman et al., 1998; Morice-Picard et al., 2016). However, the molecular link between HERC2 and neurodevelopment is still illusive.

Overall, defective ASPM, HERC2, and BRCA1 function can result in neurodevelopmental disorders. We know that the HERC2-BRCA1 axis regulates HR repair in the DNA damage response, and that inhibition of ASPM expression results in downregulated BRCA1 protein levels (Zhong et al., 2005). We were thus motivated to study whether and how ASPM is involved in the DNA damage response.

## Results

### ASPM is recruited to UV laser-induced DNA damage stripes

**Figure 1:**
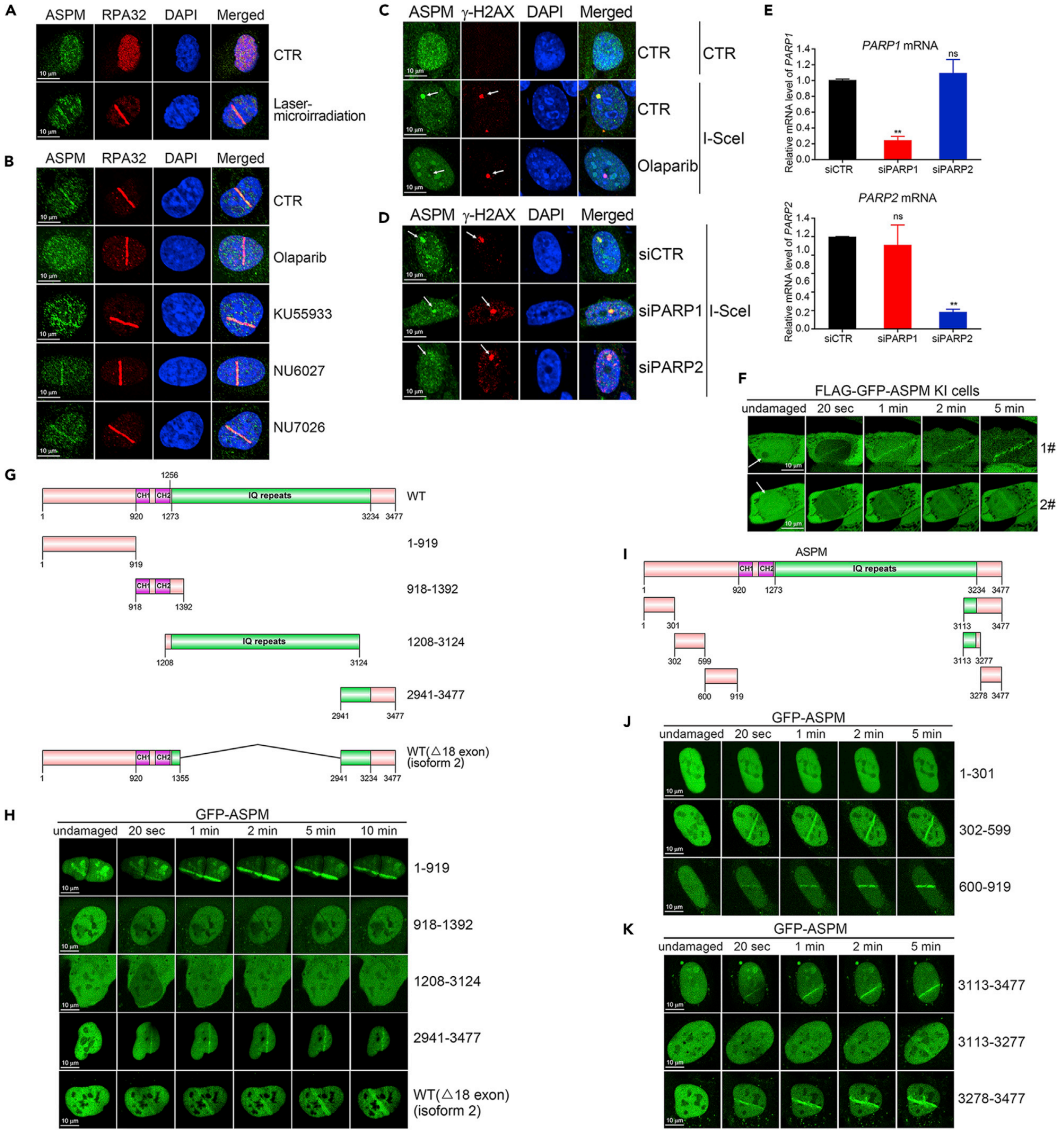
ASPM is recruited to DNA damage stripes (A) U2OS cells were subjected to UV laser-microirradiation, followed by immunofluorescence with antibodies against ASPM and RPA32. (B) U2OS cells were pretreated with a PARP inhibitor Olaparib, ATM inhibitor KU55933, ATR inhibitor NU6027 or DNA-PKcs inhibitor NU7026 for 2 hr before UV laser irradiation, followed by immunofluorescence with antibodies against ASPM and RPA32. (C) DR-U2OS cells were transiently infected with an I-SceI lentivirus for 48 hr and then subjected to immunofluorescence with antibodies against ASPM and γ-H2AX. The white arrows indicate the I-SceI induced DSB site. (D and E) DR-U2OS cells were transfected with siRNAs against PARP1 or PARP2 for 24 hr, then infected with an I-SceI lentivirus for another 48 hr before analysis by immunofluorescence with the indicated antibodies (D). The siRNA mediated knockdown efficacy of PARP1 and PARP2 was quantified by RT-PCR (E). The data represent the means of three independent experiments; data are represented as mean ± SD. p values are as follows: ∗P < 0.05, ∗∗P < 0.01, ∗∗∗P < 0.001. (F) FLAG-GFP-ASPM KI HeLa cells were exposed 365-nm UV laser irradiation and GFP fusion protein recruitment to the DNA damage sites was captured every 10 s after irradiation. (G and I) The domain structure of the ASPM isoforms and truncations. (H, J, and K) GFP-tagged ASPM fragments were expressed in U2OS cells that were subsequently irradiated with UV laser. Recruitment to laser-induced DSB sites was monitored every 10 s.

Inhibition of ASPM expression leads to a decrease in BRCA1 protein levels (Zhong et al., 2005). Given that BRCA1 is essential for DSB signaling and HR-mediated repair, we sought to determine if ASPM is involved in the cellular response to DSBs. We did not detect obvious ASPM focus formation after staining U2OS, HeLa, and HCT116 cells exposed to bleomycin, cisplatin, etoposide, or X-ray irradiation (data not shown). However, we did detect enrichment of endogenous ASPM co-localized with RPA32 in U2OS cells at UV laser-induced DNA damage stripes (Figure 1A). This enrichment was diminished when the cells were pre-treated with the PARP inhibitor olaparib, but not ATM (KU55933), ATR (NU6027), or DNA-PK (NU7026) inhibitors (Figure 1B). ASPM also co-localized with γH2AX at a single DSB induced by endonuclease I-SceI expression in DR-U2OS cells, in which a single copy of I-SceI recognition sequence was integrated into the cellular genome (Figure 1C). Again, this enrichment was diminished by pre-treatment with Olaparib (Figure 1C). Given that most cellular PARylation is contributed by PARP1 and PARP2, it was found that inhibition of PARP2 expression, but not PARP1 expression by siRNA, reduced ASPM enrichment at the I-SceI-induced DSB focus (Figures 1D and 1E).

To exclude the possibility of cross-reactivity by the anti-ASPM antibodies, we generated ASPM knock-in (KI) HeLa cells by CRISP-Cas9 technology. Specifically, we knocked in an FLAG-GFP tag prior to the *ASPM* transcriptional start site (Figure S1A). Genotyping by polymerase chain reaction (PCR) identified two positive clones of ASPM KI cells (Figure S1B). FLAG-GFP-ASPM was present in the cytosol, spindle poles in metaphase, and nucleus (Figures S1C and S1D). Immunoprecipitation (anti-FLAG) followed by immunoblotting (anti-GFP) identified both ASPM isoforms in the FLAG-GFP-ASPM immunocomplexes and total cell lysates (Figure S1E). FLAG-GFP-ASPM in ASPM KI cells was enriched at UV laser-induced DNA damage stripes and exhibited similar recruitment dynamics to endogenous ASPM (Figure 1F). Again, relocalization was blocked upon inhibiting PARP2, but not PARP1, expression (Figure S1F). ASPM is thus recruited to DSBs in a PARP2-dependent manner where it likely serves as a DSB signaling and/or repair factor.

We then mapped the region(s) essential for ASPM recruitment to UV laser-induced DNA damage stripes. We generated GFP-tagged expression constructs for full length ASPM (GFP-ASPM), isoform 2 of ASPM with exon 18 skipping (GFP-ASPM2), and various truncation mutants (Figure 1G). GFP-ASPM expression in U2OS cells was barely detectable, but GFP-ASPM2 was visible at DNA damage stripes within 1 min of UV irradiation and persisted for >10 min. The same findings were made for GFP-ASPM (1–919) and GFP-ASPM (2941–3477) but not GFP-ASPM (918–1392) or GFP-ASPM (1208–3124) (Figure 1H). We divided the amino terminus region (1–919) into three fragments and extreme carboxyl terminus (3113–3477) into three fragments (Figure 1I). We ultimately revealed that the amino terminus region (302–919) and the extreme carboxyl terminus (3278–3477) were sufficient for ASPM recruitment to DSB lesions (Figures 1I–1K). Thus, most of the IQ repeats encoded by exon 18 and the tandem CH domains are not required for ASPM relocalization onto DNA lesions, while both the amino terminus (302–919) without obvious domains/motifs and the carboxyl terminus (3278–3477) containing an armadillo (ARM)-like fold (3257–3354), a binding module well suited to binding large substrates such as proteins and nucleic acids, can be recruited to the DNA lesion.

### ASPM promotes HR-mediated repair of DSBs

**Figure 2:**
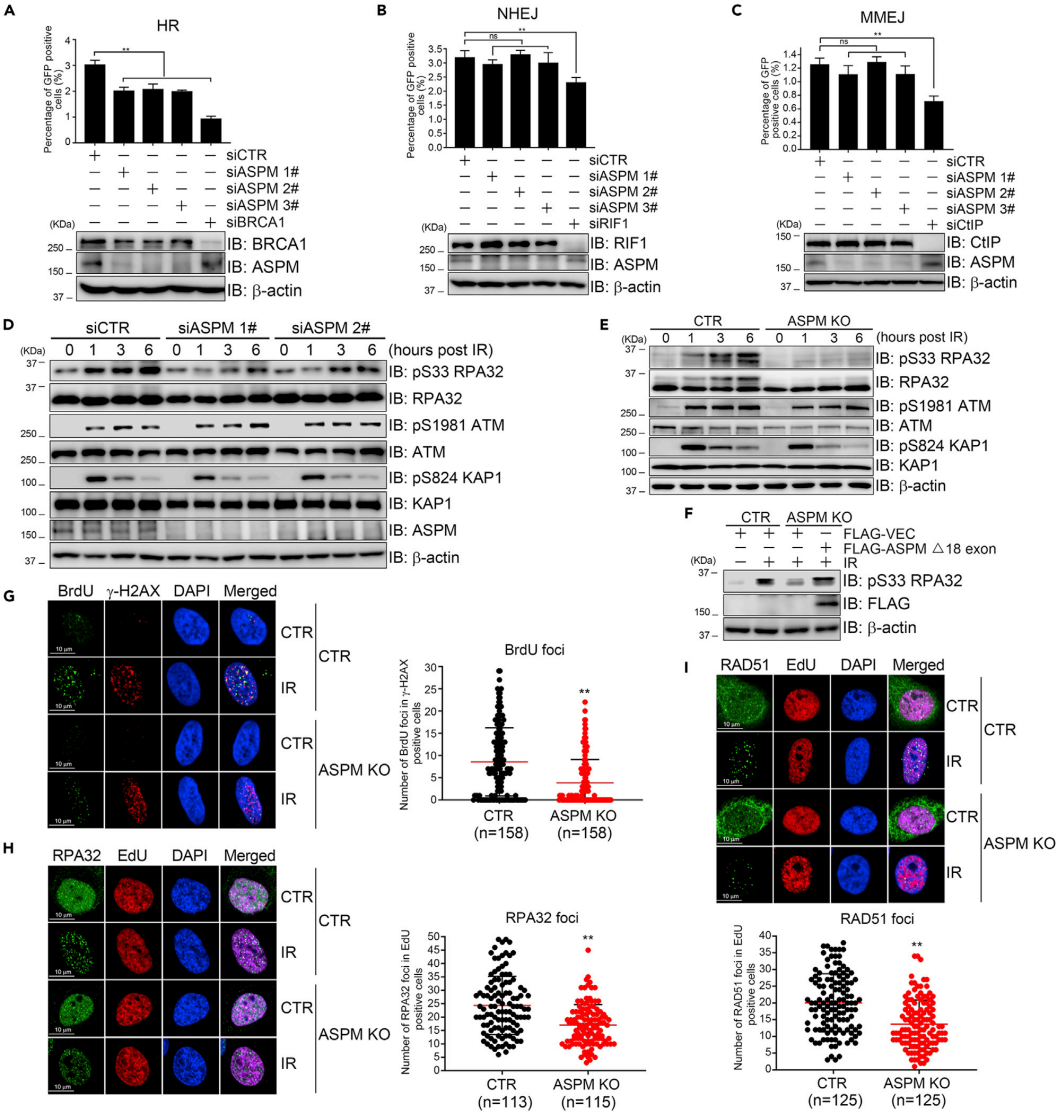
ASPM promotes HR-mediated DSB repair (A–C) DR-U2OS, EJ5-U2OS and MMEJ-U2OS cells were transfected with three different siRNA oligos targeting ASPM (siASPM1-3#), a non-targeting control siCTR, an HR positive-control siBRCA1 or an NHEJ-positive control siRIF1 or an MMEJ-positive control siCtIP for 24 hr, before infection with an I-SceI lentivirus for 48 hr and analysis of GFP-positive cells by flow cytometry. The data represent the means of three independent experiments, data are represented as mean ± SD. (D) HeLa cells were transfected with siCTR, siASPM1# or siASPM 2#. Total cell lysates were harvested 48 hr after transfection at different time points after IR treatment and analyzed by immunoblotting with the indicated antibodies. (E) ASPM KO HeLa cells were exposed to 10-Gy X-ray. The total cell lysates were harvested at different timepoints after irradiation and subjected to immunoblotting with the indicated antibodies. (F) FLAG-ASPM2 was re-expressed in ASPM KO cells and RPA32 phosphorylation after X-ray irradiation was determined. (G) WT or ASPM KO HeLa cells were incubated with BrdU for 24 hr and then exposed to 10-Gy X-ray. After 6 hr, the cells were fixed and analyzed by anti-BrdU staining to detect newly generated ssDNA during HR-mediated DNA end resection and anti-γ-H2AX. Quantification of BrdU foci in γ-H2AX positive cells is shown on the right. Data are represented as mean ± SD. (H and I) WT or ASPM KO HeLa cells were incubated with EdU for 2 hr and then exposed to 10-Gy X-ray. After 6 hr, the cells were fixed and labeled with RPA32 and RAD51 antibodies. The number of RPA32 and Rad51 foci in EdU positive cells was quantified and shown in G and H, respectively. Data are represented as mean ± SD.

Having shown ASPM enrichment onto DSBs, we next sought to determine if ASPM is involved in DSB repair. We inhibited ASPM expression using three-independent siRNA oligos transfected into HR-mediated repair reporter system DR-U2OS cells, the micro-homology end-joining (MMEJ) reporter system MMEJ-U2OS cells, and non-homologous end-joining (NHEJ) reporter system EJ5-U2OS cells, respectively. Reducing ASPM expression compromised HR-mediated DSB repair (Figure 2A) but not NHEJ (Figure 2B) or MMEJ repair (Figure 2C). Biochemically, inhibiting ASPM expression reduced RPA32 phosphorylation at S33 without impacting on ATM/KAP1 activation after 10-Gy X-ray irradiation (Figure 2D) or etoposide treatment (Figure S2A).

To exclude the possibility of off-target effects, we generated *ASPM* knockout (KO) HeLa cells, targeting exon 4 by CRISPR-Cas9 technology. We identified one clone with a premature stop codon after Phe691 (Figures S2B and S2C). Immunoblotting and immunofluorescence staining with anti-ASPM showed loss of an ASPM signal in cell compartments derived from these ASPM KO cells (Figures S2D and S2E). As expected, RPA32 phosphorylation at S33 was reduced in ASPM KO cells after 10-Gy X-ray irradiation (Figure 2E) or etoposide treatment (Figure S2F) but could be rescued upon re-expression FLAG-ASPMΔexon18 in ASPM KO cells (Figure 2F). BrdU labeling in cultured cells for 24 hr renders its incorporation into the daughter strand of DNA, thus single-strand DNA generated during the DSB end resection can be recognized by the BrdU antibody. EdU (5-Ethynyl-2′-deoxyuridine) is a thymidine analog incorporated into the DNA of dividing cells, and it can serve as a marker for newly synthesized DNA and the S/G2 phase of the cell cycle. The number of BrdU foci in γH2AX-positive cells (Figure 2G) and the number of RPA32 or RAD51 foci in EdU-positive cells (Figures 2H and 2I) after X-ray irradiation in ASPM KO cells was significantly decreased when compared to control parental cells. We made similar findings in siASPM-treated cells (Figures S2G–S2I). These findings suggest that inhibition of ASPM expression compromises DSB end resection.

### ASPM protects BRCA1 from proteasomal degradation

**Figure 3:**
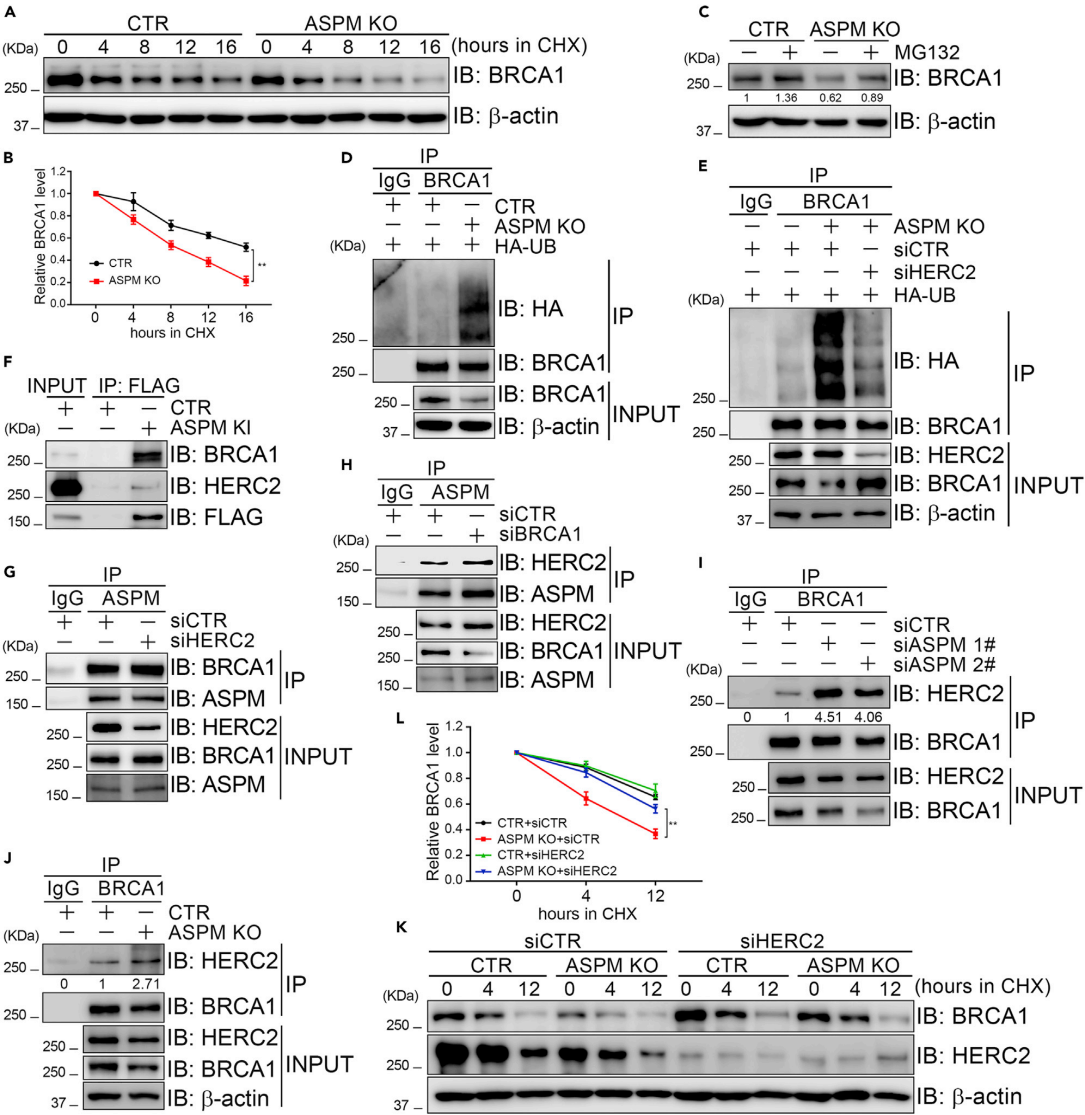
ASPM interacts with and stabilizes BRCA1 protein (A and B) ASPM KO HeLa cells and parental HeLa cells (CTR) were harvested at different time points in CHX (50 μg/mL) treatment. Total cell lysates were extracted and subjected to immunoblotting with the indicated antibodies (A), and the BRCA1/β-actin ratio was determined (B). Data are represented as mean ± SD. (C) ASPM KO HeLa cells and CTR cells were treated with MG132 (10 μM) for 4 hr. Then, total cell lysates were harvested and subjected to immunoblotting with the indicated antibodies. (D) ASPM KO HeLa cells and CTR cells were transfected with HA-UB. Total cell lysates were harvested 48 hr later and subjected to immunoprecipitation with anti-BRCA1 followed by immunoblotting with the indicated antibodies. (E) ASPM KO HeLa cells were transfected with HA-UB and control siRNA (siCTR) or HERC2 siRNA (siHERC2). Total cell lysates were harvested 48 hr later and subjected to immunoprecipitation with anti-BRCA1 followed by immunoblotting with the indicated antibodies. (F) Total cell lysates derived from FLAG-GFP-ASPM KI HeLa cells or CTR HeLa cells were subjected to immunoprecipitation with anti-FLAG, followed by immunoblotting with the indicated antibodies. (G) 293T cells were transfected with siHERC2 for 48 hr. Total cell lysates were harvested and subjected to immunoprecipitation with anti-ASPM followed by immunoblotting with the indicated antibodies. (H) 293T cells were transfected with siBRCA1 for 48 hr. Total cell lysates were harvested and subjected to immunoprecipitation with anti-ASPM followed by immunoblotting with the indicated antibodies. (I and J) Total cell lysates derived from siCTR cells and siASPM 293T cells (I), ASPM KO HeLa cells and CTR HeLa cells (J) were subjected to immunoprecipitation with anti-BRCA1 followed by immunoblotting with the indicated antibodies. (K and L) ASPM KO HeLa cells and CTR HeLa cells were transfected with siCTR or siHERC2 for 48 hr followed by CHX (50 μg/mL) treatment for the indicated durations. Total cell lysates were harvested and subjected to immunoblotting with the indicated antibodies (K), and the BRCA1/β-actin ratio was determined (L). Data are represented as mean ± SD.

Inhibiting ASPM expression by siRNA reduces BRCA1 protein levels (Zhong et al., 2005) but not mRNA levels (Figures S3A and S3B). When we treated siASPM or ASPM KO cells with CHX to inhibit protein translation, we observed significantly accelerated BRCA1 protein turnover (Figures 3A, 3B, and S3C). Meanwhile, treating these cells with MG132 to inhibit the proteasome resulted in stabilized BRCA1 protein levels (Figure 3C) and an increase in BRCA1 ubiquitination levels compared to parental cells (Figures 3D and S3D). These data imply that ASPM stabilizes BRCA1 protein stability by preventing BRCA1 ubiquitination.

The E3 ligase HERC2 interacts with BRCA1 and promotes its proteasomal degradation (Wu et al., 2010). Indeed, we found that by inhibiting HERC2 expression by siRNA in both ASPM KO cells and siASPM-depleted cells, BRCA1 ubiquitination levels could be markedly reduced, while its protein levels increased in total cell lysates (Figures 3E and S3D). We thus aimed to determine how ASPM interferes with the HERC2-BRCA1 cascade. Co-immunoprecipitation assays revealed that both endogenous HERC2 and BRCA1 were present in the FLAG-GFP-ASPM immunocomplex in FLAG-GFP-ASPM KI cells (Figure 3F). Inhibiting HERC2 expression did not alter the BRCA1 levels present in the ASPM immunocomplex (Figure 3G), and inhibiting BRCA1 expression did not alter the HERC2 levels present in the ASPM immunocomplex either (Figure 3H). However, inhibiting ASPM expression (by siRNA or ASPM KO) markedly increased the HERC2/BRCA1 ratio in the BRCA1 immunocomplex (Figures 3I and 3J). Furthermore, treating ASPM KO cells with CHX promoted BRCA1 turnover, while inhibiting HERC2 expression restored BRCA1 turnover (Figure 3K). Taken together, these results demonstrate that ASPM prevents BRCA1 from proteasomal degradation by blocking its access to HERC2.

### ASPM promotes cell survival and chromosome stability

**Figure 4:**
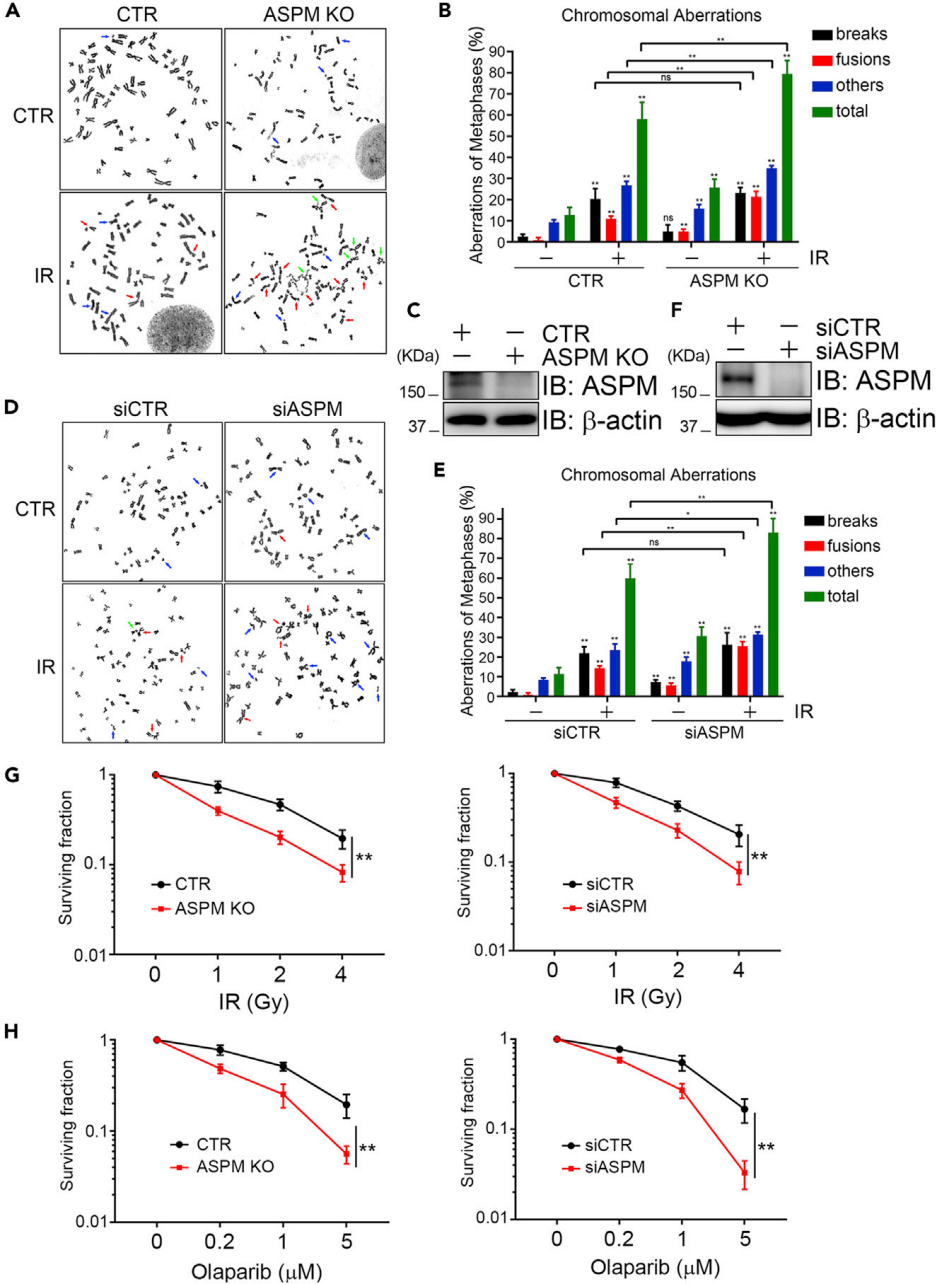
ASPM ensures chromosome stability (A–C) ASPM KO HeLa cells and CTR HeLa cells were exposed to 5 Gy X-ray irradiation for 6 hr followed by colchicine treatment. The cells were harvested prepared for Giemsa staining. More than 100 mitotic chromosomes were randomly analyzed. Representative metaphase spreads (A) and the percentages of spreads containing aberrant chromosomal structures (breaks, fusions and others) are shown (B). The arrows indicate chromosome aberrations. Data are represented as mean ± SD. The ASPM KO protein level was detected by immunoblotting (C). (D–F) HeLa cells were transfected with siCTR or siASPM for 48 hr. The transfectants were subjected to the same procedure described in (A-C). Data are represented as mean ± SD. (G) ASPM KO HeLa, CTR HeLa, siASPM HeLa, and siCTR HeLa cells were exposed to the indicated doses of X-ray irradiation (IR) and continued to grow for 14 days. The colonies were stained with crystal violet solution. The clonogenic efficiency was calculated by normalizing each group to vehicle treatment group. The data represent the means of three independent experiments; data are represented as mean ± SD. (H) ASPM KO HeLa, CTR HeLa, siASPM HeLa, and siCTR HeLa cells were treated with the indicated doses of Olaparib for 14 days. A clonogenic assay was performed as described in (G). The data represent the means of three independent experiments; data are represented as mean ± SD.

Given that ASPM promotes HR-mediated repair DSB, we hypothesized that inhibiting ASPM expression would increase chromosome instability. Indeed, mitotic spread analysis revealed that, under unperturbed conditions, chromosome fusions and total chromosomal aberrations in ASPM KO cells (Figures 4A–4C) or siASPM cells (Figures 4D–4F) significantly increased compared to control parental cells, while the increase of DNA breaks in siASPM cells, but not in ASPM KO cells, was significant likely due to the adaptation in the ASPM stable knockout cell line; under X-ray irradiation, chromosome fusions and total chromosomal aberrations, but not DNA breaks, in ASPM KO cells (Figures 4A–4C) or siASPM cells (Figures 4D–4F) significantly increased compared to control parental cells. Furthermore, inhibition of ASPM expression (by siRNA or in ASPM KO cells) resulted in cellular sensitization to X-ray irradiation (Figure 4G).

Finally, we explored if inhibiting ASPM expression exhibits synthetic lethality with PARP inhibitor. Both ASPM KO cells and siASPM-depleted cells became significantly more sensitive to Olaparib treatment in a dose-dependent manner compared to control cells (Figure 4H). Taken together, we conclude that ASPM promotes chromosome stability and might thus serve as a therapeutic target for synthetic lethality in combination with PARP inhibitors.

## Discussion

ASPM is a mitotic spindle protein (Kouprina et al., 2005; Zhong et al., 2005) that together with katanin regulates microtubule disassembly at spindle poles (Jiang et al., 2017). Here, we have uncovered a novel function for ASPM in facilitating HR repair of DSBs. We observed that inhibition of ASPM expression increases the interaction between BRCA1 and its E3 ligase HERC2 (Figures 3I and 3J), leading to enhanced ubiquitination of BRCA1 and decreased protein levels (Figure 3E), both of which are partially rescued by co-depletion of HERC2 (Figure 3E). We thus believe that ASPM serves as a scaffold regulator between HERC2 and BRCA1, blocking HERC2 from accessing BRCA1 and thus ensuring BRCA1 stability. Several groups have reported that MCPH1 promotes HR repair as well. First, the interaction between MCPH1 and Condensin II is important for efficient HR repair (Wood et al., 2008). Second, MCPH1 interacts with the transcription factor E2F1 and promote its loading on the promoter of *BRCA1* to increase the BRCA1 expression (Yang et al., 2008). Last, it has been reported that MCPH1 helps localization of BRCA2 to sites of DSBs and enhances the stability of RAD51-ssDNA filaments, both of which are key players in HR repair (Wu et al., 2009; Chang et al., 2020). Thus, ASPM and MCPH1 regulate BRCA1 levels at post-translational stage and transcriptional stage, respectively, in HR repair. Given that HR repair mainly occurs in late S/G2 phase of the cell cycle and we did not find obvious impact of inhibition of ASPM expression on NHEJ repair (Figure 2B), it is likely that ASPM/MCPH1-mediated DSB repair is late S/G2 phase-specific.

To our surprise, we observed that ASPM recruitment to DNA lesions is PARP2-dependent. PARP1 and PARP2 share a common C-terminal catalytic domain, but PARP1 possesses three zinc fingers and a BRCT domain and a WD repeat (WDR) domain (for DNA binding) at its N terminus for binding to DNA, while PARP2 has one WDR domain but lacks the zinc fingers and BRCT domain (Azarm and Smith, 2020). These differences permit PARP1 and PARP2 to bind to different DNA substrates. Furthermore, PARP2 directly binds to PAR synthesized by PARP1 at DNA lesions and promotes its enzymatic activity toward branched PAR-chain synthesis (Chen et al., 2018). We thus considered it important to explore if ASPM is an enzymatic substrate of PARP2 and/or a binding partner for branched PAR chains.

ASPM may regulate symmetric division of neural progenitor cells by modulating Cyclin E ubiquitination (Capecchi and Pozner, 2015) and/or by recruiting citron Rho-interacting kinase, which is encoded by MCPH17 (Gai et al., 2016). ASPM functions redundantly with CDK5RAP2 (MCPH3) in spindle pole focusing (Tungadi et al., 2017). Mouse *Aspm* genetically interacts *Wdr62* (WDR-containing protein 62, MCPH2) to control brain size and direct WDR62 localization, and both proteins help localize CENPJ/MCPH6 to the centrosome (Jayaraman et al., 2016). CEP152 (MCPH9) serves as a scaffold to recruit PLK4 and CENPJ to the centrosome (Cizmecioglu et al., 2010). STIL (MCPH7) interacts with CENPJ and is required for the centrosomal localization of SASS6 (MCPH14) (Vulprecht et al., 2012). All of these proteins, which are encoded by MCPH genes, genetically and biochemically interact with other and sequentially load onto the centrosomes. A *Cenpj* mutant mouse model suggests that microcephaly could be caused by mitotic delay and apoptosis (Insolera et al., 2014). *Aspm*-deficient cerebellar granule neuron progenitors show progenitor attrition with increased DNA damage and apoptosis, which could be rescue by co-deletion of *Aspm* and the apoptosis regulator p53 in medulloblastoma (Williams et al., 2015). Conditional *Brca1* ablation specifically in the dorsal telencephalon after embryonic day (E) 9.5 demonstrated that *Brca1* is required for the cerebral cortex to develop to normal size by preventing the apoptosis of early cortical progenitors (Pulvers and Huttner, 2009; Pao et al., 2014). Thus, BRCA1 instability induced by ASPM deficiency may promote p53-mediated apoptosis in neural progenitors, leading to microcephaly.

An improper response to DNA damage is a cancer hallmark and contributes to tumorigenesis and chemo/radiotherapeutic resistance. ASPM overexpression has been observed in various cancers (Xu et al., 2019, 2020; Zhou et al., 2020; Alsiary et al., 2014; Bruning-Richardson et al., 2011; Saleh et al., 2020; Xie et al., 2017; Tian and Wang, 2020; Timaner and Shaked, 2020; Hsu et al., 2019; Wang et al., 2013; Tang et al., 2019; Lin et al., 2008; Yuan et al., 2020; Wu et al., 2021) and thus might serve as a prognostic marker in various contexts. ASPM is a positive regulator of the canonical Wnt/β-catenin signaling (Major et al., 2008; Hsu et al., 2019) and thus promotes cancer stemness and progression in prostate cancer (Pai et al., 2019) and hepatocellular carcinoma (Liao et al., 2020). Based on this information, and combined with our new data, we posit that ASPM could be a promising therapeutic target in cancers characterized by ASPM-overexpression. Indeed, inhibiting ASPM expression results in an increase in DNA damage and sensitization of both immortalized cells and tumor cells to ionizing irradiation (IR) (Kato et al., 2011). Here, we have shown that inhibiting ASPM expression sensitizes cancer cells to ionizing radiation and confers a synthetic lethality effect when combined with PARP inhibitor treatment (Figure 4). We thus propose that, pending confirmation *in vivo*, dual ASPM and PARP inhibition could prove to be a precise and synergistic therapeutic strategy in ASPM-overexpressing cancers.

### Limitations of the study

In this study, we examined the role of ASPM as a safeguard of BRCA1 stability in HR repair. Our work examined the PARP2-dependent recruitment of ASPM at DSBs in response to DNA damage. It would be highly interesting to decipher the molecular actions of PARP2-mediated ASPM recruitment at DSBs.

## STAR★METHODS

### Key resources table

**Table:** 

REAGENT or RESOURCE	SOURCE	IDENTIFIER
**Antibodies**		

ASPM	BETHTL	Cat# BL1615
RPA32	BETHTL	Cat# A300-244A; RRID: AB_185548
Phospho-RPA32 (Ser33)	BETHTL	Cat# A300-246A; RRID: AB_2180847
KAP1	BETHTL	Cat# A700-014; RRID: AB_2765283
Phospho-KAP1 (Ser824)	BETHTL	Cat# A700-013; RRID: AB_2765282
HA	BETHYL	Cat# A190-208A; RRID: AB_67466
BRCA1	BETHTL	Cat# A301-377A; RRID: AB_937735
ATM	BETHTL	Cat# A300-299A; RRID: AB_263415
Phospho-ATM (Ser1981)	Abcam	Cat# ab81292; RRID: AB_1640207
HERC2	Abcam	Cat# ab85832; RRID: AB_1925057
RPA32	Abcam	Cat# ab2175; RRID: AB_302873
CtIP	Santa Cruz Biotechnology	Cat# sc-271339; RRID: AB_10608728
BRCA1	Santa Cruz Biotechnology	Cat# sc-6954; RRID: AB_626761
Rad51	Santa Cruz Biotechnology	Cat# sc-377467
GFP	Santa Cruz Biotechnology	Cat# sc-9996; RRID: AB_627695
BrdU	BD Biosciences	Cat# 347580; RRID: AB_400326
Phospho-Histone H2A.X (Ser139)	Cell Signaling Technology	Cat# 80312; RRID: AB_2799949
FLAG	Sigma	Cat# F1804; RRID: AB_262044
β-actin	Sigma	Cat# A5441; RRID: AB_476744
γ-tubulin	ABclonal Science	Cat# A9657; RRID: AB_2863751
α-tubulin	ABclonal Science	Cat# AC013; RRID: AB_2768340
Rabbit Control IgG	ABclonal Science	Cat# AC005; RRID: AB_2771930
Mouse Control IgG	ABclonal Science	Cat# AC011; RRID: AB_2770414
Peroxidase AffiniPure Goat anti-mouse lgG (H+L)	Jackson ImmunoResearch	Cat# 115-035-166; RRID: AB_2338511
Peroxidase AffiniPure Donkey anti-rabbit IgG (H+L)	Jackson ImmunoResearch	Cat# 711-035-152; RRID: AB_10015282
Fluorescein (FITC)-AffiniPure Goat Anti-Rabbit IgG (H+L)	Jackson ImmunoResearch	Cat# 111-095-003; RRID: AB_2337972
Fluorescein (FITC)-AffiniPure Goat Anti-Mouse IgG (H+L)	Jackson ImmunoResearch	Cat# 115-095-003; RRID: AB_2338589
Alexa Fluorescein 594-AffiniPure Donkey Anti-Mouse IgG (H+L)	Jackson ImmunoResearch	Cat# 715-585-150; RRID: AB_2340854
Alexa Fluorescein 594-AffiniPure Donkey Anti-Rabbit IgG (H+L)	Jackson ImmunoResearch	Cat# 711-585-152; RRID: AB_2340621

**Chemicals, peptides, and recombinant proteins**		

KU-55933	Selleckchem	Cat# S1092
KU-6027	Selleckchem	Cat# S7114
NU-7026	Selleckchem	Cat# S2893
Olaparib	Selleckchem	Cat# S1060
Etoposide	Selleckchem	Cat# S1225
MG132	Selleckchem	Cat# S2619
Chcloheximide	Selleckchem	Cat# S7418
Polyethyleneimine	Polysciences	Cat# 23966
Lipofectamine-RNAiMAX Transfection Reagent	Thermo Scientific	Cat# 13778150

**Critical commercial assays**		

Protein A SEPHAROSE	General Electric	Cat# 17061801
Protein G SEPHAROSE	General Electric	Cat# 17078001
FLAG M2 Affinity Gel	Sigma	Cat# A2220; RRID: AB_10063035
YF-594/647A Click-iT EdU Imaging Kits	US EVERBRIGHT INC.	Cat# C6017/C6018
2X SYBR Green qPCR Master Mix	BIMAKE	Cat# B21202
Total RNA Kit I	Omega Biotechnology	Cat# R6834-02
HiScript II 1st Strand cDNA Synthesis Kit (+gDNA wiper)	Vazyme Biotech	Cat# R212-02

**Experimental models: Cell lines**		

HeLa	ATCC	Cat# CCL-2
HEK293T	ATCC	Cat# CRL-3216
ASPM Knockout (ASPM KO) cell line	This paper	N/A
FLAG-GFP-ASPM Knock-in (ASPM KI) cell line	This paper	N/A

**Oligonucleotides**		

DNA primers used for qPCR analysis (see section “RT-PCR”)	N/A	N/A
RNA Oligonucleotides used for RNA interference (see section “RNA interference”)	N/A	N/A
Single-guide RNA used for CRISPR-Cas9 (see section “FLAG-GFP-ASPM knock in and ASPM knock out”)	N/A	N/A

**Software and algorithms**		

Fusion	Andor	N/A
iQ Live Cell Imaging Software 3	Andor	N/A
Image J	National Institutes of Health	https://imagej.nih.gov/
FlowJo	Treestar	https://www.flowjo.com/
GraphPad Prism 6.0	GraphPad	https://www.graphpad.com/

### Resource availability

#### Lead contact

Further information and requests for resources may be addressed to the lead contact, Xingzhi Xu, PhD at xingzhi.xu@szu.edu.cn.

#### Materials availability

All materials used in this study will be made available upon request without any restrictions.

#### Data and code availability

Further information about data supporting the conclusions of this manuscript will be made available by the lead contact to any qualified researcher.

### Experimental model and subject details

#### Cell lines and cell culture

Human U2OS cells, DR-U2OS cells (for HR) expressing an HR-mediated DSB repair reporter system (Pierce et al., 1999), EJ5-U2OS cells expressing an NHEJ-mediated DSB repair reporter system (Bennardo et al., 2008), MMEJ-GFP U2OS cells expressing an MMEJ-mediated DSB repair reporter system (Wang et al., 2012), 293T and HeLa cells were cultured in a 37°Chumidified incubator with 5% CO_2_ in DMEM (HyClone, SH30022.01) supplemented with 10% fetal bovine serum (PAN, ST30-3302) and 1% penicillin/streptomycin (HyClone, SV30010).

### Method details

#### Antibodies and chemical reagents

All ASPM fragments were sub-cloned into a pEGFP-C1 vector. Olaparib (S1060), Etoposide (S1225), MG132 (S2619), Cycloheximide (S7418), NU6027 (S7114), KU55933 (S1092) and NU7026 (S2893) were purchased from Selleck. Rabbit anti-ASPM(BL1615), anti-RPA32 (A300-244A), anti-pKAP1 S824 (A700-013), anti-KAP1 (A700-014), anti-BRCA1 (A301-377A), anti-pRPA32 S33 (A300-246A) and anti-ATM (A300-299A) antibodies were purchased from Bethyl. Rabbit anti-pATM S1981 (ab81292) and anti-HERC2 (ab85832) were purchased from Abcam. Anti-BRCA1 (sc-6954) and anti-CtIP (sc-271339) was purchased from Santa Cruz, anti-BrdU (347580) from BD Biosciences, and anti-FLAG M2 (F1804), anti-FLAG® M2 Affinity Gel (A2220) and anti-β-actin (A5441) from Sigma.

#### FLAG-GFP-ASPM knock in and ASPM knock out

Both FLAG-GFP in-frame knock in (KI) and *ASPM* knock out (KO) cell lines were generated by CRISPR-Cas9 (Clustered Regularly Interspaced Palindromic Repeats/CRISPR-associated protein 9) genome editing technology. The resulting cell lines were designated as FLAG-GFP-ASPM KI and ASPM KO, respectively. For ASPM KO, an *ASPM*-specific single-guide RNA (sgASPM: GACTGTGCCACAGCGATAAT) was inserted into the pSpCas9(BB)-2A-Puro vector (PX459) (Addgene, 62988). HeLa cells were transfected with PX549 (sgASPM) to introduce a single double-strand break (DSB) within the ASPM exon 4. The transfectants were treated with puromycin (1 μg/mL) for 2 days; then, re-seeded cells with different dilution factors were left to grow for a further 15 days before the identification and recovery of single-cell colonies. For FLAG-GFP-ASPM KI, a single-guide RNA (sgRNA: TCGAATCTGCCATGGCGAAC) was inserted into pSpCas9(BB)-2A-GFP (PX458) (Addgene, 48138). Gibson Assembly was used to assemble pDsRed N1 vector-based FLAG-GFP donor plasmids (add upstream and downstream 800 bp around the DSB site, respectively). HeLa cells were co-transfected with PX458 (sgASPM) and RFP-FLAG-GFP donor plasmids to introduce a DSB before ASPM exon 1. Both GFP- and RFP-positive single cells were selected by flow cytometry 2 days after transfection. Single clones were further confirmed by PCR and sequencing for the correct product flanking FLAG-GFP within the ASPM locus, immunofluorescence (co-localization of GFP-ASPM and α-tubulin) and immunoprecipitation (IP with anti-FLAG, IB with anti-GFP).

#### Laser-microirradiation and ionizing radiation

U2OS and HeLa cells were grown on a glass bottom cell culture dish (NEST, 801002) and then irradiated with a 365 nm UV laser (60% laser output, 16 Hz pulse) generated from a Micropoint System (Andor). Images were captured every 10 sec under a DragonFly confocal imaging system (Andor). The fluorescence images were analyzed using ImageJ (NIH). For ionizing radiation (IR), HeLa cells were exposed to 1.6 Gy/min generated by a Radsource RS-2000pro X-Ray irradiator.

#### RNA interference

The following siRNA oligonucleotide duplexes were used: ASPM 1# (5’ GUGGUGAAGGUGACCUUUCdTdT3’); ASPM 2# (5’AUGCUAACAAGCAGGUUAAUU-dTdT-3’); ASPM 3# (5’CACUCGUCAUUCAGAAAUAdTdT-3’); HERC2 (5’ GCGGAAGCCUCAUUAGAAAdTdT-3’), BRCA1 ( 5’ CUAGAAAUCUGUUGCUAUGdTdT-3’) , RIF1 (5’-AGACGGUGCUCUAUUGUUAdTdT-3’), CtIP (5’ GCUAAAACAGGAACGAAUCdTdT-3’), PARP1 (5’-CAAAGUAUCCCAAGA AGUUdTdT-3’); PARP2 (5’-CUAUCUGAUUCAGCUAUUAdTdT-3’). All siRNAs were transfected into cells using Lipofectamine™ RNAiMAX (Invitrogen, 13778150).

#### RT-PCR

Total RNA was extracted using an RNA isolating Kit (Foregene) according to the manufacturer’s instructions. Then, 1 μg total RNA was used for cDNA synthesis with random hexamers. The following primers were used in the RT-PCR amplification: PARP1-F: CGGAGTCTTCGGATAAGCTCT, PARP1-R: TTTCCATCAAACATGGGCGAC, PARP2-F: GCCTTGCTGTTAAAGGGCAAA, PARP2-R: TCCTTCACAATACACATGAGCC, ASPM-F: TGCAGTGGGTGAACATGAAAA, ASPM-R: CGAAGAGGGTGTTACCTCGTTT, BRCA1-F: TTGTTACAAATCACC CCTCAAGG, BRCA1-R: CCCTGATACTTTTCTGGATGCC. For the initial amplification, the PCR proceeded with a denaturation step at 95°C for 10 min, followed by 28 cycles of denaturation at 95°C for 1 min, primer annealing at 55°C for 30 s, and primer extension at 72°C for 45 s, before a final extension at 72°C for 5 min. Real-time PCR was carried out using an ABI PRISM 7500 Sequence Detection System (Applied Biosystems). The housekeeping gene β-actin (F: CATGTACGTTGCTATCCAGGC, R: CTCCTTAATGTCACGCACGAT) was used as an internal control.

#### Immunoprecipitation and immunoblotting

Immunoblotting and immunoprecipitation were performed as previously described (Xu and Stern, 2003).

#### Immunofluorescence, EdU and BrdU labeling

For immunofluorescence, U2OS and HeLa cells were first micro-irradiated with a MicroPoint System (Andor) or exposed to IR as described above. Both cell types were washed with PBS and then fixed with 4% paraformaldehyde (PFA) at room temperature (RT) for 5 min, permeabilized with Triton-X100 (0.5%) for 5 min, and blocked with 2% BSA (in 0.1% PBST) for 30 min at RT. The cells were incubated with primary antibody for 1 h, and then washed three times with PBST before being incubated with a fluorescent-conjugated secondary antibody for a further 1 h. The cells were then stained with DAPI for 2 min and images were captured using a DragonFly confocal imaging system (Andor).

For BrdU incorporation (ssDNA staining), HeLa ASPM KO or knock down cells were cultured with BrdU (10 μM) for 24 h. The cells were then exposed to 10 Gy IR and then permeabilized with Triton-X100 (0.5%) for 3 min, fixed with 4% PFA for 10 min and blocked with 2% BSA (in 0.1% PBST) for 30 min all at RT. After washing with PBS, the cells were incubated with an anti-BrdU antibody for 1 h followed by three washes with blocking buffer before a FITC-conjugated secondary antibody was added and incubated for 1 h. For EdU labeling (S/G2 phase), HeLa ASPM KO or knock down cells were cultured with EdU (10 μM) for 2 h and then exposed to 10 Gy IR. After 6 h, the cells were treated according to the procedure described in the YF®647A Click-iT EdU Imaging Kit (US EVERBRIGHT INC, C6018).

#### DSB repair assay

DR-U2OS (HR) and EJ5-U2OS (NHEJ) cells, containing a single copy of a DR-GFP or EJ5-GFP reporter gene (respectively) integrated into the genome were used for DSB repair assays. The assays were performed as previously described (Pierce et al., 1999). MMEJ-GFP U2OS cells, in which a full-length EGFP cassette is inactivated as a result of a 27-bp oligonucleotide insertion containing an I-SceI cleavage site flanked by a 9-bp microhomology sequence, were used to monitor microhomology-mediated end joining-mediated DSB repair (Wang et al., 2012). All three reporter cells were infected with an I-SceI lenti-virus for 48 h before analysis by flow cytometry (Beckman, CytoFlex).

#### Chromosome aberrations assay

Metaphase chromosome analysis was performed as previously described (Huertas and Jackson, 2009). Briefly, ASPM KO or knock down HeLa cells were exposed to 5 Gy IR and then treated with colchicines (0.4 μg/ml) for 6 h before harvesting. The collected cells were incubated in a hypotonic solution (75 mM KCl) for 30 min, then fixed in a 3:1 methanol/acetic acid solution before storage at −20°C overnight. The cells were then dropped onto slides, incubated for 2 h at 60°C and Giemsa-stained. Images were captured under a DragonFly confocal imaging system (Andor). More than 100 mitotic chromosomes were randomly selected and analyzed.

#### Cell survival assay

ASPM KO or knock down HeLa cells were split into 6-well plates at a density of 150 cells per well. The cells were exposed to 0, 1, 2, 4 Gy IR or 0, 0.25, 1, 5 mM Olaparib and then incubated for 14 days. The surviving colonies were fixed and stained with crystal violet.

### Quantification and statistical analysis

All experiments were repeated three times independently. All statistical analyses were performed in Microsoft Excel and GraphPad Prism 8. A two-tailed non-paired Student’s t-test was used to determine significant differences between two treatment groups. A p value <0.05 was considered statistically significant. ∗p < 0.05, ∗∗p < 0.01, ∗∗∗p < 0.001; ns, not significant.
