# Supplementary material for: ASPM promotes homologous recombination-mediated DNA repair by safeguarding BRCA1 stability
Source: iScience. 2021 May 12;24(6):102534. doi: 10.1016/j.isci.2021.102534 (PMC8184511; doi:10.1016/j.isci.2021.102534)
Supplement: Document S1. Figures S1–S3 [file mmc1.pdf]

iScience, Volume *24*

Supplemental information

ASPM promotes homologous recombination-mediated DNA repair by safeguarding BRCA1 stability

Shibin Xu, Xingxuan Wu, Peipei Wang, Sheng-Li Cao, Bin Peng, and Xingzhi Xu

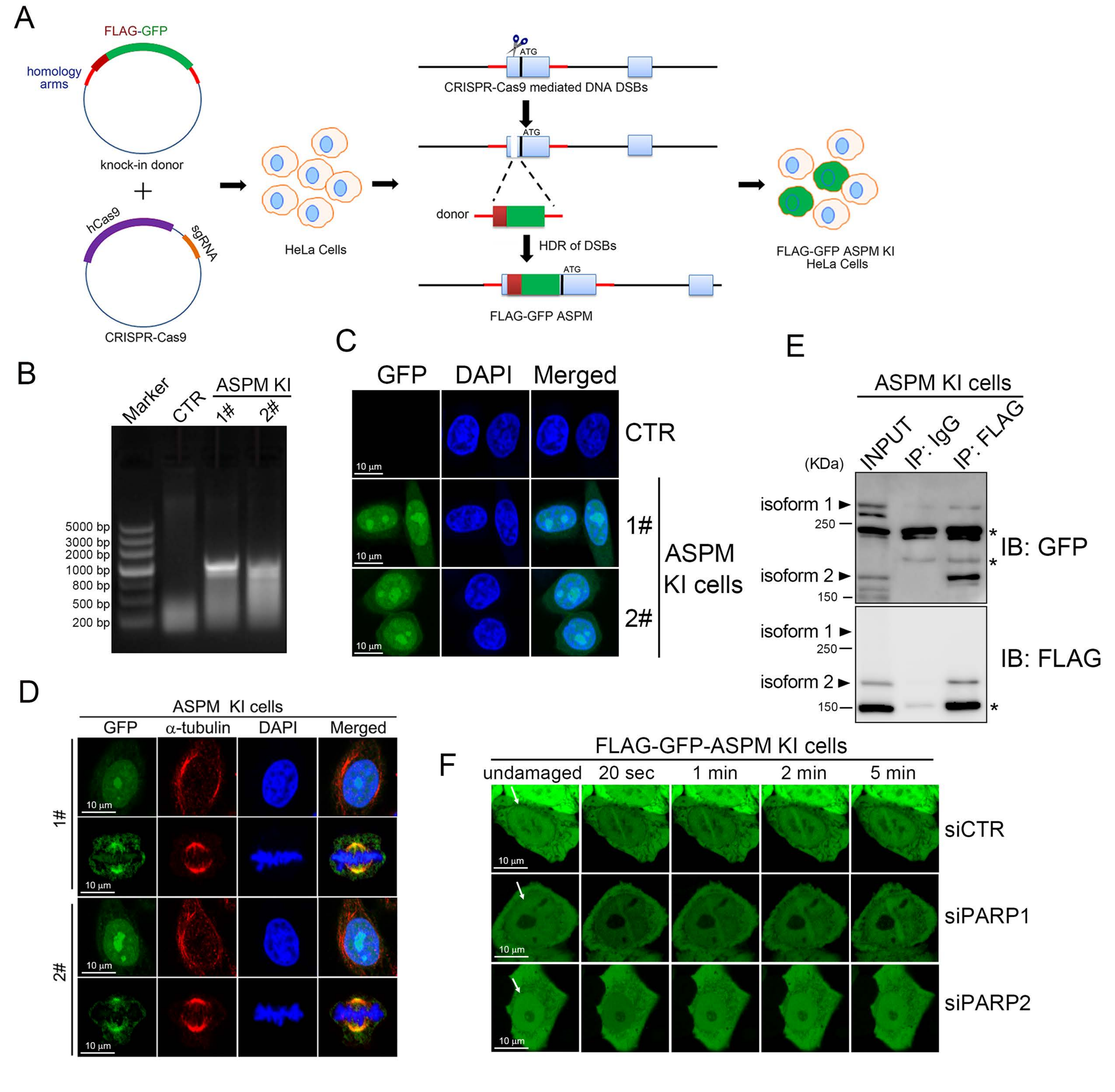
A
FLAG-GFP
homology arms
knock-in donor
+
hCas9
sgRNA
CRISPR-Cas9
HeLa Cells
ATG
CRISPR-Cas9 mediated DNA DSBs
donor
HDR of DSBs
FLAG-GFP ASPM
FLAG-GFP ASPM KI HeLa Cells
B
Marker
CTR
ASPM KI
1#
2#
5000 bp
3000 bp
2000 bp
1000 bp
800 bp
500 bp
200 bp
C
GFP
DAPI
Merged
CTR
1#
2#
ASPM KI cells
10 μm
E
ASPM KI cells
INPUT
IP: IgG
IP: FLAG
(KDa)
isoform 1
250
isoform 2
150
IB: GFP
IB: FLAG
D
ASPM KI cells
GFP
α-tubulin
DAPI
Merged
1#
2#
F
FLAG-GFP-ASPM KI cells
undamaged
20 sec
1 min
2 min
5 min
siCTR
siPARP1
siPARP2

Figure S1. ASPM is recruited to DNA damage stripes. Related to Figure 1.

(A) Schematic overview of the FLAG-GFP-ASPM knock-in (KI) in HeLa cells that was generated by CRISPR-Cas9 technology. A 3xFLAG-GFP tag was inserted in frame into the position right before the first ATG codon.

(B-D) FLAG-GFP-ASPM KI cells were verified by PCR (B), and the subcellular localization (C), and mitotic spindle localization (D) was checked by immunofluorescence.

(E) Total cell lysates derived from FLAG-GFP-ASPM KI HeLa cells were subjected to immunoprecipitation with an anti-FLAG antibody followed by immunoblotting with the indicated antibodies. The *asterisk* indicates non-specific signals.

(F) FLAG-GFP-ASPM KI HeLa cells were transfected with siCTR, siPARP1, or siPARP2 for 48 h before UV laser irradiation. The white arrows indicate the starting point and direction of irradiation.

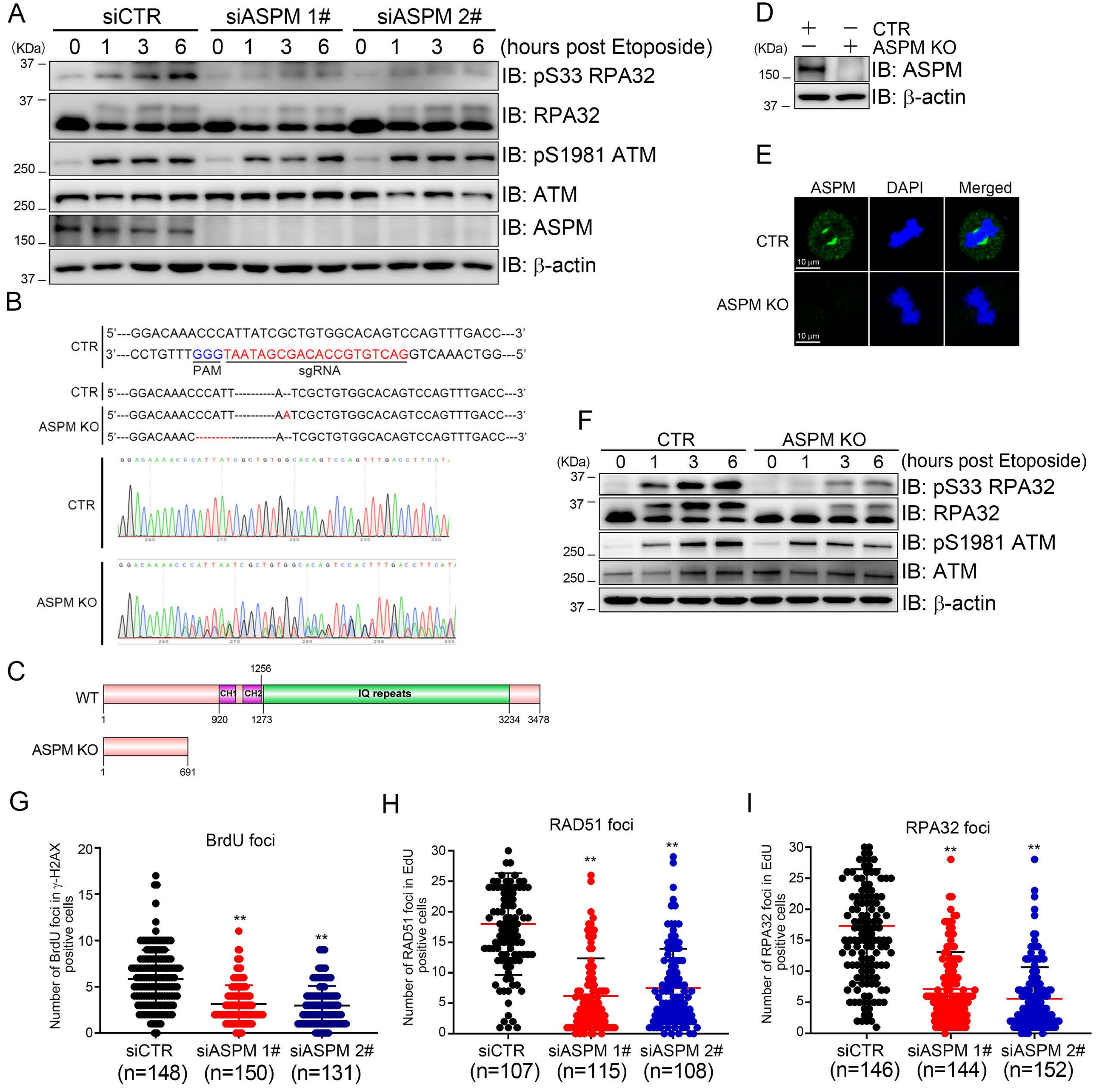
A
siCTR
siASPM 1#
siASPM 2#
(KDa)
0 1 3 6 0 1 3 6 0 1 3 6 (hours post Etoposide)
37
IB: pS33 RPA32
37
IB: RPA32
250
IB: pS1981 ATM
250
IB: ATM
150
IB: ASPM
37
IB: β-actin
B
CTR
5'---GGACAAACCCATTATCGCTGTGGCACAGTCCAGTTTGACC---3'
3'---CCTGTTTGGGTAATAGCGACACCGTGTCAGGTCAAACTGG---5'
PAM
sgRNA
CTR
5'---GGACAAACCCATT----------A--TCGCTGTGGCACAGTCCAGTTTGACC---3'
ASPM KO
5'---GGACAAACCCATT----------AATCGCTGTGGCACAGTCCAGTTTGACC---3'
5'---GGACAAAC--------------------A--TCGCTGTGGCACAGTCCAGTTTGACC---3'
CTR
ASPM KO
C
WT
CH1
CH2
IQ repeats
1256
1
920
1273
3234
3478
ASPM KO
1
691
D
+ − CTR
(KDa) − + ASPM KO
150
IB: ASPM
37
IB: β-actin
E
ASPM
DAPI
Merged
CTR
ASPM KO
10 μm
10 μm
F
CTR
ASPM KO
(KDa)
0 1 3 6 0 1 3 6 (hours post Etoposide)
37
IB: pS33 RPA32
37
IB: RPA32
250
IB: pS1981 ATM
250
IB: ATM
37
IB: β-actin
G
BrdU foci
Number of BrdU foci in γ-H2AX positive cells
20
15
10
5
0
**
**
siCTR (n=148)
siASPM 1# (n=150)
siASPM 2# (n=131)
H
RAD51 foci
Number of RAD51 foci in EdU positive cells
30
25
20
15
10
5
0
**
**
siCTR (n=107)
siASPM 1# (n=115)
siASPM 2# (n=108)
I
RPA32 foci
Number of RPA32 foci in EdU positive cells
30
25
20
15
10
5
0
**
**
siCTR (n=146)
siASPM 1# (n=144)
siASPM 2# (n=152)

Figure S2. ASPM promotes HR-mediated DSB repair. Related to Figure 2.

(A) siCTR HeLa cells or siASPM HeLa cells were treated with etoposide for 1 h, washed with PBS for three times, and replenished with fresh medium. Total cell lysates were harvested at different time points after treatment and subjected to immunblotting with antibodies as indicated. (B) Verification of ASPM KO HeLa cells by sequencing.

(C) Schematic of the truncated ASPM peptides, the constructs of which were transfected into ASPM KO HeLa cells.

(D and E) Verification of ASPM KO HeLa cells by immunoblotting with anti-ASPM (D) and mitotic spindle localization (E).

(F) ASPM KO HeLa and CTR HeLa cells were treated as described in (A).

(G-I) siCTR HeLa cells and siASPM HeLa cells were as described in Figures 2G, 2H and 2I. Data are represented as mean ± SD. P values are as follows: $*p < 0.05$, $**p < 0.01$, $***p < 0.001$.

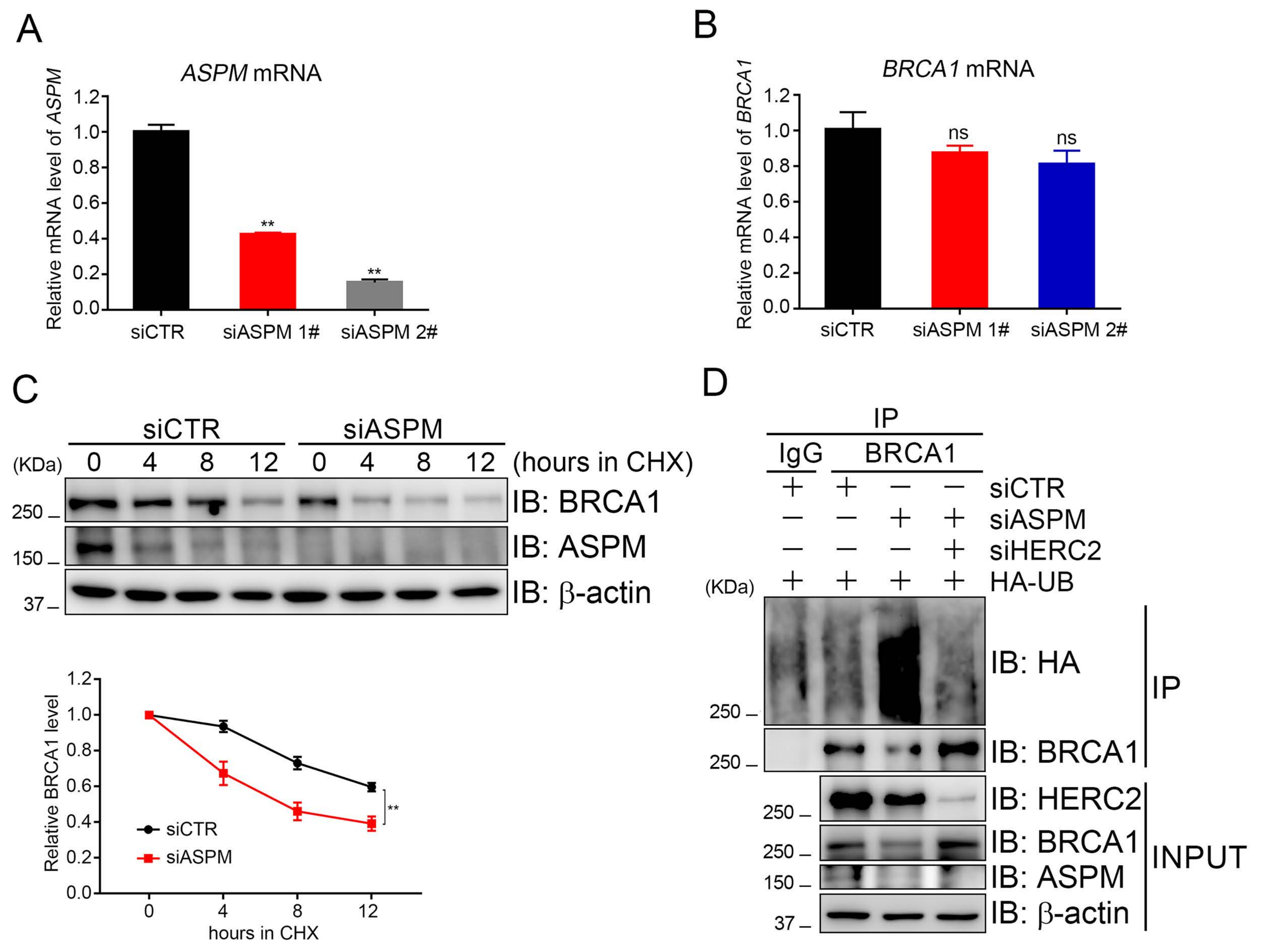

A
ASPM mRNA
Relative mRNA level of ASPM
1.2
1.0
0.8
0.6
0.4
0.2
0.0
**
**
siCTR
siASPM 1#
siASPM 2#
B
BRCA1 mRNA
Relative mRNA level of BRCA1
1.2
1.0
0.8
0.6
0.4
0.2
0.0
ns
ns
siCTR
siASPM 1#
siASPM 2#
C
siCTR
siASPM
(KDa)
0 4 8 12 0 4 8 12
(hours in CHX)
250
150
37
IB: BRCA1
IB: ASPM
IB: β-actin
Relative BRCA1 level
1.2
1.0
0.8
0.6
0.4
0.2
0.0
0
4
8
12
hours in CHX
**
siCTR
siASPM
D
IP
IgG
BRCA1
siCTR
siASPM
siHERC2
HA-UB
(KDa)
250
250
250
250
150
37
IB: HA
IB: BRCA1
IP
IB: HERC2
IB: BRCA1
IB: ASPM
IB: β-actin
INPUT

Figure S3. ASPM interacts with and stabilizes BRCA1 protein. Related to Figure 3.

(A) ASPM mRNA level in siASPM HeLa cells. The data represent the means of three independent experiments, data are represented as mean ± SD.

(B) BRCA1 mRNA level in siASPM HeLa cells. The data represent the means of three independent experiments, data are represented as mean ± SD.

(C) siCTR HeLa cells and siASPM HeLa cells were harvested at different time points in CHX (50 mg/ml) treatment. The total cell lysates were extracted and subjected to immunoblotting with the indicated antibodies before the BRCA1/b-actin ratio was quantified. Data are represented as mean ± SD.

(D) siCTR HeLa cells and siASPM HeLa cells were transfected with HA-UB and control siRNA (siCTR) or HERC2 siRNA (siHERC2). The total cell lysates were harvested and subjected to immunoprecipitation with anti-BRCA1 followed by immunoblotting with the indicated antibodies.
